# Leveraging the HIV response to strengthen pandemic preparedness

**DOI:** 10.1371/journal.pgph.0001511

**Published:** 2023-01-24

**Authors:** Chris Collins, Michael T. Isbell, Quarraisha Abdool Karim, Annette H. Sohn, Chris Beyrer, Allan Maleche

**Affiliations:** 1 Friends of the Global Fight Against AIDS, Tuberculosis and Malaria, Washington, D.C., United States of America; 2 Independent Consultant, New York, NY, United States of America; 3 Centre for the AIDS Programme of Research in South Africa, Durbin, South Africa; 4 Department of Epidemiology, Mailman School of Public Health, Columbia University, New York, NY, United States of America; 5 TREAT Asia, amfAR, Bangkok, Thailand; 6 Duke Global Health Institute, Durham, NC, United States of America; 7 Kenya Legal and Ethical Issues Network on HIV and AIDS, Nairobi, Kenya; Tony Blair Institute for Global Change, UNITED KINGDOM

## Abstract

The COVID-19 pandemic and the expectation of future pandemic threats have generated a global dialogue on strengthening pandemic preparedness and response (PPR). Thus far, this dialogue has largely failed to fully consider the critical role that established, disease-specific programs played in national and regional COVID-19 responses, and the potential for these programs to contribute to stronger pandemic preparedness for the future. The HIV response is an important example of a global health initiative that is already making substantial contributions to PPR. Both the infrastructure and core principles of the HIV response have much to contribute towards pandemic preparedness that is more effective and equitable than seen in the response to COVID-19. This review examines how HIV-related resources and principles can support communities and countries in being better prepared for emerging disease threats, with a specific focus on evidence from the COVID-19 pandemic. Drawing on the current literature, the review explores the clear, multi-faceted intersection between the HIV response and the central elements of pandemic preparedness in areas including surveillance; supply chain; primary care; health care workforce; community engagement; biomedical research; universal access without discrimination; political leadership; governance; and financing. There are many opportunities to be more strategic and purposeful in leveraging HIV programs and approaches for preparedness. Avoiding the longstanding temptation in global health to create new siloes, PPR initiatives, including the new Pandemic Fund at the World Bank, should invest in and build out from existing programs that are already making health systems more inclusive and resilient, including the global response to HIV.

## Introduction

We are living in an “age of pandemics” [1]. By the end of 2021, the COVID-19 pandemic had caused an estimated 15 million deaths worldwide [2] and triggered the most severe global economic downturn since the Great Depression [3]. COVID-19 highlighted humanity’s interconnectedness and revealed how ill-prepared we were as a global community to respond to sudden health and socioeconomic threats. COVID-19 was preceded by numerous other disease threats, including a severe outbreak of Ebola in western Africa in 2014-2016 [4] and a subsequent outbreak in the Democratic Republic of Congo [5]; the SARS outbreak of 2003 [6]; a 2009-2010 outbreak of H1N1 influenza virus [7]; and the Middle East respiratory syndrome coronavirus (MERS-CoV) [8]. Most recently, outbreaks of monkeypox, which has been endemic in several countries in Africa and Latin America, have occurred in high-income countries with only a limited history of monkeypox transmission [9].

There is growing attention to the importance of pandemic prevention, preparedness and response (PPR). A recent high-level review of the COVID-19 response cites “multiple failures of international cooperation” and issued several proposals to strengthen preparedness [10]. In 2022, the board of directors of the World Bank approved the creation of the Pandemic Fund for PPR [11]. The World Health Assembly has launched a process to develop a treaty or other instrument to strengthen PPR responses [12], and the World Health Organization has established a pandemic preparedness hub in Berlin [13].

Thus far, the global dialogue on PPR, primarily focused on establishing new structures and mechanisms, has largely failed to fully consider the critical role that established, disease-specific programs played in national and regional COVID-10 responses, and the potential for these programs to contribute to stronger pandemic preparedness for the future. A comprehensive and holistic approach to strengthening PPR would take advantage of synergies with existing systems and build on lessons learned from prior pandemic responses. It would also adopt approaches, such a trust building, community engagement, and a commitment to leaving no one behind, which are central to the success of any public health initiative.

Both the infrastructure and core principles of the HIV response have much to contribute towards strengthening PPR. Since it was first recognized in the early 1980s, HIV has infected more than 84 million people and caused more than 40 million deaths [14]. At its height, the HIV epidemic caused unprecedented declines in life expectancy across sub-Saharan Africa [15]. HIV elicited a response that was arguably unique in the annals of global health – prioritizing human rights and gender equality, welcoming the leadership of communities and people living with HIV, drawing on principles of global solidarity and shared responsibility to mobilize unprecedented financial resources, building on inclusive health governance, and aiming for universal access to the fruits of scientific research [16–18].

The global HIV response has achieved substantial gains, with the number of people newly infected with HIV declining by more than half since the epidemic’s peak in the mid-1990s and AIDS-related deaths falling by nearly two-thirds since 2004 [19]. Still, the effort remains unfinished. Primary prevention of HIV infection is inadequate, with marginalized populations, often with limited access to services, representing roughly two-thirds of all new infections [19].

The HIV response is an important example of a global health initiative that is already making substantial contributions to PPR [20]. HIV-financed laboratories, clinical service sites, health personnel and surveillance systems rapidly adapted to support national COVID-19 efforts, and the leadership of the HIV response swiftly engaged to aid COVID-19 responses [20]. The HIV response experienced considerable difficulties and disruptions during COVID-19, with especially serious consequences for HIV testing and HIV prevention services, but innovation and resilience among HIV stakeholders enabled the response to minimize these disruptions [21]. Although it proved adaptable in the face of the most widespread global health crisis in a century, the HIV response nevertheless lost momentum as a result of COVID-19, which it is now working to regain [22]. Health care providers and community actors in the HIV response, while ultimately vital to the resilience of HIV services in the context of COVID-19, struggled in the early stages of the COVID-19 pandemic with shortages of personnel, personal protective equipment and working linkages with clinical services [23].

Robust pandemic preparedness will not be achieved merely by creating a new fund or enacting a new treaty. While working to mobilize new resources for PPR, decision-makers should strategically leverage existing programs – including HIV programming - that help build secure, resilient and prepared health systems.

This review examines how HIV resources and approaches can support communities and countries in being better prepared for emerging disease threats, with a specific focus on evidence from the COVID-19 pandemic. It outlines key elements of PPR alongside aspects of the HIV response. In the final section, we note opportunities for health decision-makers and stakeholders to be more intentional and strategic about leveraging the HIV response to strengthen PPR capacity.

## Core elements of PPR and potential synergies with the HIV response

Taking account of lessons learned from the COVID-19 response, several panels of experts have identified key elements of PPR [1, 24, 25]. These exercises have identified both technical and institutional imperatives for improved preparedness. There is a clear, multi-faceted intersection between the HIV response and the central elements of PPR.

### Surveillance and laboratories

Surveillance systems that are vigilant and globally networked are essential to effective PPR [1] – as reflected in the World Bank’s identification of “surveillance, collaborative intelligence and early warning” as key funding priorities for PPR funding [25]. HIV investments have markedly strengthened public health surveillance and information systems, especially in sub-Saharan African countries with a heavy burden of HIV and tuberculosis [26]. These surveillance systems, built in large measure through HIV-focused investments, proved pivotal to national efforts to monitor and respond to COVID-19 [20, 27], and can be built upon to serve broader health systems and preparedness needs.

During COVID-19, laboratories built or strengthened through HIV investments, including more than 3,000 supported by the U.S. President’s Emergency Plan for AIDS Relief (PEPFAR), quickly pivoted to diagnose and monitor COVID-19 [28]. Molecular surveillance, including phylogenetics established for advancing understanding of HIV transmission dynamics, proved to be an important platform to monitor SARS-CoV-2 mutations and notably the emergence of variants of concern. Botswana used its HIV laboratory infrastructure to develop a monitoring strategy for COVID-19, providing the world with advance warning in late 2021 of the emergence of the Omicron variant [29]. In addition to strengthening surveillance systems, the HIV response has also driven innovation in methods to maximize the strategic use of surveillance data for public health, including priority setting and monitoring the impact of interventions [30]. With considerable investments through the HIV response, national health surveillance systems have become more systematized, sophisticated, and granular in the data they collect [30] – key characteristics that are essential to pandemic vigilance and response.

As human, technological and financial resources are finite, it is inevitable that HIV-financed laboratory systems and staff will struggle when saddled with additional responsibilities, especially during a health crisis as serious and widespread as COVID-19. Although HIV-related lab services largely delivered during COVID-19, the difficult choices posed by the added burdens associated with the COVID-19 pandemic underscore that further investments in building laboratory capacity and healthcare personnel in resource-limited settings is an urgent priority with respect to PPR [31].

### Health systems and primary care

Resilient, adaptable health systems are essential in the event of a new pandemic. Aspects of primary health care that are important for pandemic preparedness include prevention, detection and treatment. HIV programming can be a bridge to stronger, more inclusive health systems and primary care that also advance preparedness.

PEPFAR and the Global Fund to Fight AIDS, Tuberculosis and Malaria (Global Fund) each invests more than US$ 1 billion annually to strengthen health systems in low- and middle-income countries [28, 32]. This health infrastructure rapidly adapted to serve as the front line of the COVID-19 response in many countries [20, 28]. HIV programming charted an innovative path in health care delivery, emphasizing a public health approach, patient centered care, task shifting, and community involvement in service provision [33, 34]. Extensive evidence demonstrates that HIV programming can strengthen primary care, improve outcomes, promote efficiency and broaden health services for clients [35–37]. The patient centered approach that has characterized HIV care can be effectively leveraged to address other health conditions, such as cardiovascular disease [38].

Health care workers are essential to health systems that are robust, people-centered and resilient, yet the world faces an acute shortage of health workers, primarily in low- and middle-income countries [39]. The World Bank Pandemic Fund will reportedly prioritize initiatives to strengthen “health workforce capacities” [25]. Here, too, the HIV response is already making major contributions. PEPFAR alone has trained nearly 300,000 health care workers and supports the operations of 70,000 clinics in low- and middle-income countries [40]. These health workers and systems have been associated with improved outcomes across a broad array of health domains, including cardiovascular and other non-communicable diseases [16, 36, 41–43]. During the COVID-19 pandemic, PEPFAR advanced task-sharing approaches to encourage multifunctionality of health service delivery teams and systems and also repurposed staff to ensure both the continuity of HIV services and the delivery of COVID-19 related testing, treatment and vaccination services [44].

In addition to public and private sector health service systems, effective pandemic responses depend on robust and engaged community systems [45], which play an especially important role in reaching remote, marginalized and underserved populations, who are often most heavily affected by pandemics. For key populations, integration of HIV services with broader preparedness functions may be more appropriate within community-centered systems that are specifically designed to address their needs [42, 46–48], even as work continues to transform health services to be more inclusive and person-centered.

### Community engagement, health communication and trust

Community trust emerged as a critical element in COVID-19 responses. An analysis of COVID-19 response success factors recommended greater emphasis on “risk communication and community engagement strategies” in addressing future pandemics [49]. In a subsequent correspondence, the study authors noted the value of “investing in community engagement strategies to respond to the specific needs and concerns of marginalized subgroups” [50]. Reviews of the international health regulations and other evaluations have identified “risk communication” as a key PPR gap [25]. This gap became particularly visible during the COVID-19 response, as shifting public health advice, lack of trust in public health authorities, and the broad circulation of misinformation and disinformation impeded COVID-19 control efforts [51]. Effective risk communications requires extensive, ongoing community engagement, which has been identified as one of the core domains the PPR Pandemic Fund will support [25].

The HIV response has remained in the vanguard of global efforts to promote community engagement and leadership to improve health outcomes [35]. Communities engaged in the HIV response advocate for sound public policies and robust investments, deliver services to populations often unreached by facility-based approaches, and play a key watchdog role by monitoring services systems and national responses [35]. When COVID-19 emerged, community-led HIV responses served as first responders, rapidly innovating to preserve HIV service access during national lockdowns and to contribute to national COVID-19 responses [27, 52]. As UNAIDS has observed of community providers, “they are pandemic response experts” [53].

The COVID-19 response has an unfortunately spotty record in taking on board lessons learnt from HIV regarding the transformative value of community engagement and community-led communications. While communities acted on their own to protect themselves during the COVID-19 and to preserve essential HIV services, COVID-19 responses often de-prioritized “bottom-up” approaches, relying on “top-down” communications strategies and frequently failing to prioritize communities in the designing, planning and governance of COVID-19 responses [45]. In the Democratic Republic of Congo, faith leaders expressed difficulties in delivering effective information about COVID-19 because these leaders were asked by the government to deliver specific, predetermined health messages and were not invited to co-develop messages appropriate for their congregations [54].

Community based responses have the potential to help drive strategic efforts to harness the HIV response for broader health needs, including preparedness. Trained, well-equipped and strategically integrated community health workers can educate community members regarding emerging health threats, diagnose infection and navigate clients to person-centered services [55]. Effectively leveraging community responses requires increased investment in community delivery systems that have established trust with their constituents [56].

### Engaging stakeholders in robust biomedical research

Strong, sustained investments in biomedical and other health-related research are key to effective PPR. This was amply demonstrated during COVID-19, when well-resourced public-private partnerships developed and began to roll out preventive vaccines in record time [57, 58].

HIV investments have driven extraordinary advances in biomedical research [59], including revolutionizing scientific understanding of the human immune system and producing highly active antiretroviral therapy, among the most important scientific discoveries of the last half-century. HIV research has not only dramatically improved the prevention and treatment of HIV itself, but has also yielded benefits for the treatment of cancer and immune-mediated diseases [60]. HIV research was the foundation on which successful efforts to develop COVID-19 vaccines were based [61], and HIV clinical trial infrastructure was used for the evaluation of COVID-19 vaccines and therapeutics. However, in contrast to HIV, which has prioritized rapid, worldwide scale-up of the fruits of biomedical research, the COVID-19 pandemic saw limited commitment to global access to essential health tools – an outcome the world must avoid in responding to future pandemics.

One important characteristic of HIV research is its extensive engagement of people living with HIV, affected communities and other key stakeholders across all stages of the research process [62]. HIV investments have helped build research capacity in low- and middle-income countries [63, 64] – an important feature given recent experience with COVID-19, where comparatively few clinical trials were conducted in low- and middle-income countries [65, 66]. Biomedical research for pandemic preparedness should learn from the HIV approach, engaging end users in the design and dissemination of innovations, and expanding research capacity in low- and middle-income.

### Pharmaceutical manufacturing capacity in low- and middle-income regions

Experience during the COVID-19 pandemic underscores the need for robust pharmaceutical manufacturing capacity in all regions in the event of a pandemic, as shortages of personal protective equipment, diagnostics, medicines and preventive vaccines undermined the global response [24, 67]. The Independent Panel on Pandemic Prevention and Response called for urgent efforts to build vaccine manufacturing capacity in low- and middle-income regions as well as international agreements on voluntary licensing and technology transfer [24].

Mechanisms pioneered through the HIV response can help drive equitable access to PPR-related commodities in low- and middle-income countries. Using a patent pooling approach, the Medicines Patent Pool has signed voluntary license agreements with the makers of more than 30 therapeutic and preventive medicines for HIV, tuberculosis, hepatitis C and COVID-19, with sub-licenses provided to generic manufacturers across the world to enable worldwide uptake of essential health commodities [68]. Unitaid, initially created to drive innovation to address HIV, malaria and tuberculosis, supports market-shaping interventions to reduce the prices and accelerate uptake of medical innovations across a broad range of health areas [69]. Routinizing the use of voluntary licenses, technology transfer and forward-looking market-shaping interventions would strengthen the world’s resilience against future health threats.

### Universal access without discrimination

As we learned during COVID-19, pandemics are not “under control anywhere unless they are controlled everywhere” [70]. Universal, worldwide access to essential diagnostic, prevention and treatment tools is essential to pandemic responses. As with earlier pandemics and disease outbreaks, COVID-19 also underscored the dangerous effects of stigma and discrimination on the health and well-being of the most vulnerable and marginalized communities and on societies’ ability to respond effectively to pandemics [71–73].

The HIV response has brought a commitment to strive towards universal access, an outcome that is vital to the success of PPR. HIV advocates proactively and successfully worked to break the traditional paradigm for biomedical innovations, which expected low-income countries to wait decades before obtaining meaningful access to biomedical breakthroughs. Advocacy to increase flexibility in intellectual property rules helped enable wider access. In the face of considerable skepticism, the HIV response in 2003 launched perhaps the most ambitious undertaking in global health history – to achieve worldwide, universal access to the antiretroviral regimens that were slashing AIDS-related death rates in high-income countries [74]. In 2021, 75% of all people living with HIV worldwide were receiving antiretroviral therapy [19].

Non-discrimination, including in access to health services, has long served as a bedrock principle for the HIV response, which has advocated for legal reform in line with human rights principles. In 2021, 107 countries had mechanisms in place to assist people in reporting and obtaining redress for HIV-related discrimination [19]. To reach all communities effectively, preparedness services must learn from the HIV approach, prioritizing non-discrimination and efforts to fight stigma.

### Procurement and supply chain

Systems and personnel for the delivery of health commodities play a central role in responding to pandemics, and the COVID-19 response proved less successful in delivering interventions than in developing them. Supply chain deficiencies undermined responses to COVID-19, as commodities, such as personal protective equipment, treatments and vaccines were often in short supply; national lockdowns impeded procurement and distribution of commodities; and competition between countries drove the cost of commodities higher than many countries could bear.

HIV-related investments in strengthening national health commodity procurement and supply chain management [75] proved highly valuable in the response to COVID-19, enabling the procurement and shipment of oxygen, ventilators, protective equipment and laboratory equipment [28, 75]. The HIV response is a pioneer of pooled procurement of medical commodities for low- and middle-income countries [76], an approach that has been shown to lower prices, reduce delays and minimize the risk of commodity stock-outs, and which could improve commodity access in the next pandemic [77, 78].

### Global solidarity and political leadership

The emergence of COVID-19 could have been a moment for global solidarity to collectively tackle a new disease threat. Instead, many countries emphasized nationalistic approaches. The United States, the world’s largest global health donor, markedly prioritized domestic over global access to COVID-19 vaccines, initially refusing even to participate in the COVID-19 Vaccines Global Access (COVAX) initiative. The failure to act globally from the onset of the new pandemic led to stark inequities in COVID-19 vaccine and therapeutic access in low- and middle-income countries. In the case of pandemics, nationalistic approaches are also self-defeating, as no country is safe unless a pandemic is brought under control everywhere.

Advancing PPR would benefit mightily from rekindling the sense of global solidarity that gave rise to the AIDS movement. More than a quarter-century after the global AIDS movement was launched with the founding of the Joint United Nations Programme on HIV/AIDS (UNAIDS), the HIV response has built – and to a remarkable degree sustained – strong political support and an ethos of accountability for results. A series of high-level political declarations endorsed by the United Nations General Assembly have set clear, time-bound targets in the HIV response [79], and countries report annually on progress towards HIV commitments and targets, using standardized metrics [80]. Civil society advocacy has played a pivotal role in building and sustaining political commitment on HIV [81].

### Financing

The G20 High Level Independent Panel estimates that annual investments of US$ 15 billion will be needed over the next five years to build strong PPR capacity [1]. The World Bank and WHO estimate that at least US$ 10.5 billion in external PPR financing will be needed annually for low- and middle-income countries [25]. Domestic investments in PPR will also need to increase, as these currently account for only 1-3% of total government health outlays in low- and middle-income countries [25].

The HIV response has a proven expertise and track record in resource mobilization that PPR efforts should leverage. HIV advocates rejected “resource scarcity” or “absorptive capacity” arguments, insisting that funding should match the need. A critical aspect of this resource mobilization success has been an emphasis by PEPFAR and the Global Fund, among other programs, on showing concrete results from their investments, particularly in the number of people receiving HIV treatment and the number of lives saved [82, 83]. International funding for HIV drove a surge in official development assistance for health beginning in the 1990s [84]. Although international HIV assistance has flattened in recent years, the degree to which external support for HIV responses in low- and middle-income countries has been sustained is noteworthy [85]. Domestic financing for HIV in low- and middle-income countries has steadily increased since 2000, with especially sharp increases in domestic investments in a number of countries [52]. HIV financing already directly contributes to PPR, with a recent analysis finding that more than one-third of the Global Fund’s investments support health security [86]. Ensuring that investments are adequate to meet the need and demonstrating clear returns on investments will be key to the success of PPR efforts.

### Governance

Effective governance is essential to ensure coordination, transparency and accountability for results [1]. Global health governance has evolved in recent decades, with a greater voice in decision-making among low- and middle-income countries and more formal engagement of civil society [87]. The HIV response has been at the forefront of this evolution, offering important lessons for the emerging PPR architecture.

The HIV response has long adhered to the “three ones” principles – a single national HIV action framework, a single national HIV coordinating body, and a single system for monitoring and evaluating the HIV response [88]. When the COVID-19 pandemic emerged, countries from diverse regions and all income categories immediately drew on the expertise and experience of HIV coordinating bodies to contribute to, and in some cases lead, national responses to COVID-19 [20].

National HIV strategies and coordinating mechanisms have specifically been designed to be multisectoral and to integrate the contributions of civil society and affected communities. Country coordinating mechanisms, which are charged with developing and overseeing the implementation of Global Fund grants, include voting representation of people living with HIV, civil society and affected communities [89]. Likewise, PEPFAR has prioritized the engagement of communities in the development of country and regional operational plans that guide implementation of PEPFAR-funded HIV programs [87]. At the global level, the governing boards of the leading global initiatives spawned by the HIV response, including UNAIDS, the Global Fund, and Unitaid, each include participation by civil society and people living with HIV [87]. These inclusive approaches to HIV governance have expanded the constituency for the HIV response, united diverse stakeholders around common goals, and helped ensure that programs meet the needs of the communities and settings most heavily affected by HIV – offering a template and important lessons learned for PPR initiatives.

### First, do no harm

Building from HIV infrastructure to reinforce pandemic preparedness needs to be done intentionally and will require close monitoring of funding and outcomes to ensure that HIV programs are not compromised, particularly for key populations. Though the HIV response is a model, its success is incomplete, with 1.5 million new HIV infections annually, 10 million living with HIV still not receiving treatment and unequal access to care for marginalized individuals [19].

While the potential for the HIV response to contribute to PPR is clear, placing additional burdens on HIV programming without providing new resources will inevitably cause the quality of HIV services to suffer [46]. Even as HIV service integration is both appropriate and imperative in many settings, tailored service delivery options need to be preserved for key populations, who are frequently poorly served by mainstream systems [48]. Increasing investment in well-trained, sensitized health professionals, community health workers and community systems offers a route to build out from HIV services in a way that strengthens the HIV response while addressing additional health priorities, including preparedness and resilience [90].

The characteristics of health systems that are critical to pandemic responses – comprehensive, resilient, well-resourced, person-centred, adaptable in response to strategic data – are precisely the elements of sound health systems generally. The HIV response potentially offers a model not only for preparing for and responding to pandemics, but for the strengthening of health systems more generally.

## Policy implications for leveraging the HIV response for PPR

HIV investments are already making major contributions to PPR in low- and middle-income countries. Efforts to strengthen PPR, including through the new Pandemic Fund at the World Bank, should specifically encourage and fully leverage synergies with the PPR benefits already being yielded by the HIV response.

There are many opportunities to be more strategic and purposeful in leveraging HIV programs and approaches. Avoiding the longstanding temptation in global health to create new siloes, PPR initiatives, including the Pandemic Fund, should invest in and build out from existing programs that are already making health systems more inclusive and resilient, including programs for HIV, tuberculosis and malaria.

Both the Global Fund and PEPFAR pivoted their approaches during COVID-19, using the same laboratories, supply chains, facilities and health workers to address the new pandemic. Innovations that have contributed to the success of the HIV response, including the test-and-treat model, differentiated, community-based service models, and use of mobile or pop-up service sites, were adopted to tackle COVID-19. Both PEPFAR and the Global Fund have outlined ways in which they are poised to expand their work to strengthen pandemic preparedness [91, 92].

The HIV movement has generated new approaches to global health principles and governance. Efforts to strengthen PPR should build on these innovations. Global solidarity, universal access and non-discrimination principles should be prioritized, and civil society should be engaged and effectively resourced at all stages of building PPR capacity, including governance. Investing in robust and inclusive scientific research, including the engagement of communities at all stages of the research process, will be key to building sustainable PPR and health system resilience.

Through focused planning and program flexibility, both the Global Fund and PEPFAR can be more intentional in encouraging PPR-related benefits from HIV investments. In the case of the Global Fund, this will require new resources (including from the Pandemic Fund), personnel with the appropriate expertise, dialogues within country coordinating mechanisms on the integration of PPR into disease-specific programming, and a specific focus on synergizing the Global Fund’s health systems investments with investments by other parties. To fully leverage PEPFAR investments for PPR, PEPFAR could offer country teams and grantees more flexibility and incentives to strengthen PPR functions. This will require new resources for PEPFAR and may warrant changes in PEPFAR reauthorization language and Country Operational Planning guidance.

It will also be important to fully leverage other key actors in the HIV response. UNAIDS can continue to provide advocacy, policy leadership and normative guidance on how best to build out from the HIV response to support pandemic preparedness while safeguarding and accelerating progress on HIV. The UN has an important role to play in co-ordination and priority setting. The market-shaping innovations of Unitaid can be leveraged to promote accessibility and affordability of biomedical innovations for future pandemics. The voluntary licensing scheme of the Medicines Patent Pool should be recognized as standard operating procedure for facilitating access to health commodities to address future pandemics. Existing procurement mechanisms, such as the Global Fund’s wambo.org online procurement platform, should support regional procurement mechanisms, such as the Africa Medical Supplies Platform [93].

## Conclusion

The infrastructure and core principles that have defined the HIV response should play a catalytic role in building PPR capacity. However, in working to leverage HIV investments to strengthen PPR, it is essential these efforts not slow progress towards the global goal of ending the AIDS epidemic, especially as HIV investments have flattened and progress in preventing new HIV infections has slowed [19]. Maintaining accountability for results in the HIV response will remain critical in this new era of pandemics. In particular, building out from HIV infrastructure to support PPR must ensure meaningful, non-discriminatory access to people-centered services for key populations.

The world urgently needs to prepare for the next pandemic. With new resources that are strategically channeled, it is possible to accelerate progress against HIV while strengthening pandemic preparedness in a manner that advances global health equity and security.
